# Fatal Bleeding From a Common Iliac Arterio-Ureteral Fistula in an Older Patient

**DOI:** 10.7759/cureus.21578

**Published:** 2022-01-24

**Authors:** Ryuichi Ohta, Keita Inoue, Chiaki Sano

**Affiliations:** 1 Communiy Care, Unnan City Hospital, Unnan, JPN; 2 Urology, Unnan City Hospital, Unnan, JPN; 3 Community Medicine Management, Shimane University Faculty of Medicine, Izumo, JPN

**Keywords:** hemorrhagic shock, hematuria, iliac arterio-ureteral fistula, bleeding, anemia

## Abstract

Fatal ureteral bleeding is rare among elderly individuals. One cause of bleeding can be a fistula between the arteries and urinary organs, such as a common iliac arterio-ureteral fistula. However, the clinical presentation of fistulas can vary. As microscopic hematuria can be an initial finding, detecting the fistula without gross hematuria may be difficult. Here, we report a case of microhematuria that progressed to massive hematuria caused by a common iliac arterio-ureteral fistula. The patient was an 86-year-old man with a chief complaint of cardiopulmonary arrest. He was resuscitated in the previous condition. He had microscopic hematuria. One month later, the patient underwent rehabilitation. He was in hemorrhagic shock with massive hematuria. Further investigation revealed a right common iliac arterio-ureteral fistula. This case demonstrates the importance of investigating anemia in the elderly, including anemia of urinary origin, despite it being rare.

## Introduction

Fatal ureteral bleeding is rare among elderly individuals. Several kinds of pathophysiology cause fatal ureteral bleeding, such as arterial ruptures and fistulas with arteries [1]. Arterial injuries caused by cancers can cause critical bleeding from the kidneys, ureters, bladder, and prostate [2,3]. The most common cancer that causes bleeding is prostate cancer, as the prostate is a highly vascular organ [4]. Meanwhile, bleeding from fistulas between the arteries and urinary organs is also fatal and can be difficult to diagnose because clarifying the location of the fistula can be challenging [5].

Bleeding can be caused by fistulas between the arteries and ureters, especially among patients with ureteral stents. The common iliac arterio-ureteral fistula is one of the most common fistulas as the ureters anteriorly cross the common iliac artery [6]. The inducing factor is ureteral stents used for stenosis or obstruction of the ureter caused by cancers or stones [1,7]. Long-term installation of ureteral stents can cause inflammation, causing ulcers on the wall [3,8]. In addition, long-term stenting can cause stricture with the surrounding tissues, inducing a fistula between the common iliac artery and ureters [8].

The clinical presentation of a common iliac arterio-ureteral fistula can vary. As microscopic hematuria can be an initial finding, the susceptibility of the fistula may be difficult to assess without gross hematuria [3,8]. Here, we report a case of microhematuria that progressed to massive hematuria caused by a common iliac arterio-ureteral fistula. This case demonstrates the importance of investigating anemia in the elderly, including anemia of urinary origin, even though it can be rare.

## Case presentation

An 86-year-old man who was independent of activities of daily living came to our hospital with the chief complaint of syncope with a return of spontaneous circulation from cardiopulmonary arrest (CPA). On the admission day, he was manually emptying his neo-rectum reservoir secondary to extensive recto-sigmoid cancer resection and lost consciousness. His wife found him unconscious and made an emergency call for him to be transported by ambulance. The patient and his family noticed gross hematuria before admission. His medical history included gastric ulcer, hypertension, dyslipidemia, chronic kidney disease, asthma, chronic obstructive lung disease, and colon cancer with abdominoperineal resection of the rectum, producing an end stoma and artificial bladder because of the invasion into the bladder with bilateral ureteral stents. His medications included a fluticasone inhaler, amlodipine, simvastatin, rebamipide, and lactomin.

The initial vital signs were a body temperature of 37.7°C, blood pressure of 83/43 mmHg, a pulse of 84 beats/min, respiratory rate of 16 times/min, and SpO_2_ of 98% (oxygen 6 L). The Glasgow Coma Scale score was 14. Physical examination revealed pale conjunctiva, systolic cardiac murmur without radiation, and cold hands and legs. No other abnormalities were observed upon physical examination. The initial laboratory data revealed a hemoglobin (Hb) level of 7.0 g/dL (two months ago it was 9.2 g/dL), brain natriuretic peptide of 187.7 pg/mL, and C-reactive protein of 14.31 (Table 1).

**Table 1:** Initial laboratory data.

Marker	Level	Reference
White blood cells	7.3	3.5–9.1 × 10^3^/μL
Neutrophils	65.5	44.0%–72.0%
Lymphocytes	31.2	18.0%–59.0%
Monocytes	2.6	0.0%–12.0%
Eosinophils	0.3	0.0%–10.0%
Basophils	0.4	0.0%–3.0%
Red blood cells	2.30	3.76–5.50 × 10^6^/μL
Hemoglobin	7.0	11.3–15.2 g/dL
Hematocrit	22.1	33.4%–44.9%
Mean corpuscular volume	95.9	79.0–100.0 fL
Platelets	11.5	13.0–36.9 × 10^4^/μL
Total protein	6.8	6.5–8.3 g/dL
Albumin	2.0	3.8–5.3 g/dL
Total bilirubin	0.2	0.2–1.2 mg/dL
Aspartate aminotransferase	29	8–38 IU/L
Alanine aminotransferase	9	4–43 IU/L
Lactate dehydrogenase	262	121–245 U/L
Blood urea nitrogen	27.7	8–20 mg/dL
Creatinine	1.75	0.40–1.10 mg/dL
Estimated glomerular filtration rate	29.3	>60.0 mL/min/L
Serum Na	137	135–150 mEq/L
Serum K	2.7	3.5–5.3 mEq/L
Serum Cl	103	98–110 mEq/L
Creatinine kinase	42	56–244 U/L
C-reactive protein	14.31	<0.30 mg/dL
Urine test		
Leukocyte	+2	
Nitrite	−	
Protein	−	
Glucose	−	
Urobilinogen	−	
Bilirubin	−	
Ketone	−	
Blood	+1	
pH	7.0	
Specific gravity	1.007	
Urine red blood cells	30	High-power field
Urine white blood cells	>50	High-power field
Fecal occult blood	(−)	

**Figure 1:**
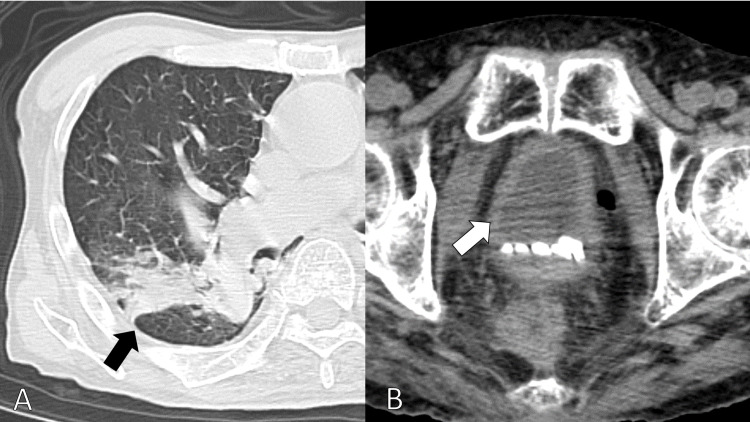
CT scan from the neck to the pelvis showing infiltration of the right lung (A). And no high-density areas in the bladder indicative of hemorrhage (B).

We performed a heart ultrasound indicating no valvular abnormalities with an ejection fraction of 0.6. A CT scan from the neck to the pelvis revealed infiltration of the right lung and no high-density areas in the colon and bladder, suggesting hemorrhage (Figure 1).

**Figure 2:**
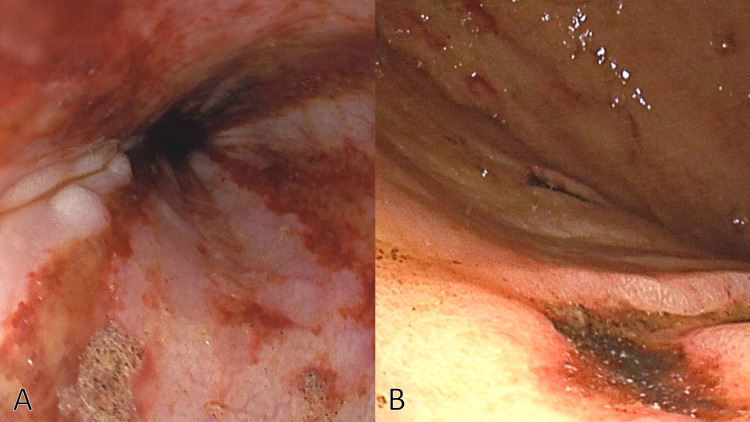
Upper gastrointestinal endoscopy revealing multiple esophageal ulcers (A) and multiple gastric ulcers (B).

The patient was diagnosed with vasovagal reflux progressing to CPA, followed by aspiration pneumonia. Upper gastrointestinal endoscopy revealed multiple esophageal and gastric ulcers (Figure 2), which were considered to be the causes of anemia.

Gram staining of the sputum revealed a large number of leukocytes and Gram-positive cocci, suggesting Streptococcus and anaerobic bacterial infections. We treated the patient with ampicillin (6 g) and sulbactam (9 g). For anemia, red blood cells were transfused, and gastric ulcers were treated with omeprazole.

His clinical course was good. After treatment, he did not require oxygen, and his vital signs were normal. He could eat and move to the portal toilet. His condition nearly returned to the previous state. His laboratory tests indicated a Hb level of 10 g/dL, and he did not experience dyspnea and palpitation during rehabilitation.

**Figure 3:**
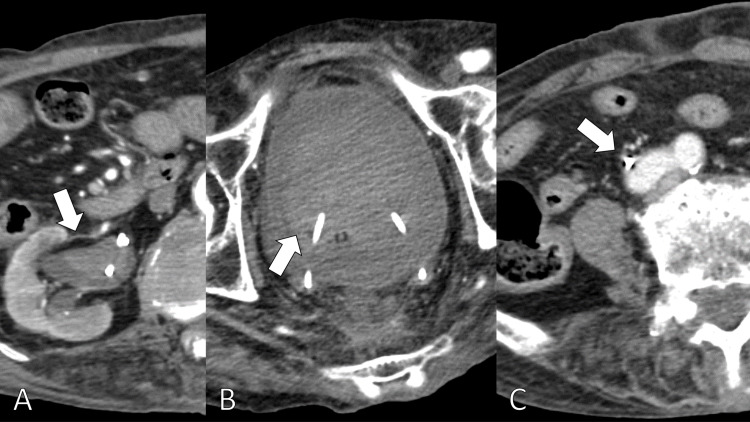
Contrast-enhanced CT revealing the higher density area in the right renal pelvis (A), higher density areas in the urinary bladder (B), and the right common iliac arterio-ureteral fistula (C).

On the 21st day from admission, he experienced lightheadedness during rehabilitation. His urine was bright red. The laboratory data revealed a Hb of 6.0 g/dL. The contrast CT revealed higher density areas in the right renal pelvis and bladder and a right common iliac arterio-ureteral fistula (Figure 3).

His condition was stabilized with noradrenaline and blood transfusion. However, his urethral bleeding was not stopped. We consulted a urologist and cardiologist for further consultation and treatment at a tertiary hospital; however, the patient and his family refused because they did not wish to leave the town and wanted palliative care at our hospital. We respected their decision and provided palliative care to the patient. Unfortunately, one week later he died.

## Discussion

This case demonstrates the challenge of diagnosing common iliac arterio-ureteral fistulas among the elderly and the importance of investigating anemia in this population, including anemia of urinary origin despite it being rare. Older patients can have multiple causes of anemia, such as gastric ulcers and common iliac arterio-ureteral fistulas. Therefore, clinicians should carefully consider various causes of anemia among older patients when anemia is not cured completely with the primary treatment for suspected disease.

Older patients tend to have several age-related diseases, which can be described as multimorbidity [9,10]. Multimorbidity can cause various symptoms, including anemia caused by chronic diseases such as diabetes and chronic renal diseases, suppressing bone marrows [11,12]. Moreover, multimorbidity can lead to polypharmacy, which pertains to the regular use of five to six medications [13]. However, polypharmacy can cause anemia, leading to admission [14,15]. In this case, the patient had various diseases and medicines causing anemia, which caused his hematuria and delayed the diagnosis of a common iliac arterio-ureteral fistula. Furthermore, the primary diagnosis of anemia caused by gastric ulcer could cause premature closure of the differential diagnosis of the patient’s anemia. Thus, having a broad differential diagnosis of anemia among the elderly is crucial.

To detect common iliac arterio-ureteral fistulas, identifying the disease and precipitating factors causing the disease should be critical for effective diagnosis and treatment. A common iliac arterio-ureteral fistula can be caused by connecting the common iliac artery and unilateral ureter [6,16]. The pathophysiology can be triggered by calcification of the common iliac artery and artificial devices in the common iliac artery or ureters [3,8]. Calcifications of the outer wall of the common iliac artery can irrigate surrounding organs, including the ureters [3,8]. Artificial devices can cause irrigation and penetration of the walls, resulting in a fistula [7]. In this case, the patient had ureteral stents and irrigation in the neighboring organs, resulting in a higher risk of a common iliac arterio-ureteral fistula. Given such medical history, clinicians should suspect the possibility of a fistula, considering the acute exacerbation of anemia.

For effectively diagnosing anemia, recognizing symptoms of anemia among older rural patients is essential. Older people tend to manage their symptoms independently without seeking help from others [17-19]. A previous study has reported that anemia in older rural patients tends to be disregarded as a problem [15]. Because of aging wherein, older people have various age-related symptoms, symptoms of anemia may be difficult to identify [20]. In this case, the patient already had anemia two months prior, although the anemia was not sufficiently assessed for appropriate treatment. In addition, older rural people may not have symptoms of anemia due to multimorbidity [12], although with effective management of symptoms, they could have a high quality of life and tend to use primary care [19]. Therefore, clinicians should be aware of older patients’ symptoms by carefully screening for subtle symptoms and abnormalities in laboratory data.

## Conclusions

Diagnosing arterio-ureteral fistulas among the elderly is challenging because the elderly can have various diseases causing anemia. To detect common iliac arterio-ureteral fistulas, identifying the disease and precipitating factors causing the disease is critical for providing effective diagnosis and treatment. Moreover, recognizing the symptoms of anemia among older rural patients is essential.
